# Social Influences on Inequity Aversion in Children

**DOI:** 10.1371/journal.pone.0080966

**Published:** 2013-12-02

**Authors:** Katherine McAuliffe, Peter R. Blake, Grace Kim, Richard W. Wrangham, Felix Warneken

**Affiliations:** 1 Department of Human Evolutionary Biology, Harvard University, Cambridge, Massachusetts, United States of America; 2 Department of Psychology, Boston University, Boston, Massachusetts, United States of America; 3 History, Anthropology, and Science, Technology, and Society Program, Massachusetts Institute of Technology, Cambridge, Massachusetts, United States of America; 4 Department of Psychology, Harvard University, Cambridge, Massachusetts, United States of America; Durham University, United Kingdom

## Abstract

Adults and children are willing to sacrifice personal gain to avoid both disadvantageous and advantageous inequity. These two forms of inequity aversion follow different developmental trajectories, with disadvantageous inequity aversion emerging around 4 years and advantageous inequity aversion emerging around 8 years. Although inequity aversion is assumed to be specific to situations where resources are distributed among individuals, the role of social context has not been tested in children. Here, we investigated the influence of two aspects of social context on inequity aversion in 4- to 9-year-old children: (1) the role of the experimenter distributing rewards and (2) the presence of a peer with whom rewards could be shared. Experiment 1 showed that children rejected inequity at the same rate, regardless of whether the experimenter had control over reward allocations. This indicates that children’s decisions are based upon reward allocations between themselves and a peer and are not attempts to elicit more favorable distributions from the experimenter. Experiment 2 compared rejections of unequal reward allocations in children interacting with or without a peer partner. When faced with a disadvantageous distribution, children frequently rejected a smaller reward when a larger reward was visible, even if no partner would obtain the larger reward. This suggests that nonsocial factors partly explain disadvantageous inequity rejections. However, rejections of disadvantageous distributions were higher when the larger amount would go to a peer, indicating that social context enhances disadvantageous inequity aversion. By contrast, children rejected advantageous distributions almost exclusively in the social context. Therefore, advantageous inequity aversion appears to be genuinely social, highlighting its potential relevance for the development of fairness concerns. By comparing social and nonsocial factors, this study provides a detailed picture of the expression of inequity aversion in human ontogeny and raises questions about the function and evolution of inequity aversion in humans.

## Introduction

The occurrence of extensive cooperation in human societies creates numerous opportunities for exploitation by free riders [1]–[3]. In order to avoid being exploited, individuals must regulate their contributions to cooperative endeavors by attending to their payoffs relative to those of social partners. In line with this reasoning, human adults show a strong aversion to inequitable payoff distributions, i.e. they sacrifice personal gain in order to avoid inequity [4]. For example, in the ultimatum game, people often reject allocations of resources that place them at a disadvantage relative to a partner (i.e. disadvantageous inequity), preferring nothing to a small relative reward [5]. This behavior violates rational choice models that predict that people should accept any non-zero offer of a desirable resource [6]. More surprisingly, in some situations adults also reject advantageous allocations in which they receive more than a peer (advantageous inequity) [4], [7]–[8]. Despite some variation, an aversion to unequal resource distributions has been established in a wide variety of cultural communities [9]–[11], demonstrating the apparent ubiquity of inequity aversion across human populations.

Research on children and nonhuman animals demonstrates that inequity aversion is not restricted to human adults. Studies of children show that sensitivity to inequity is an important feature of early development [12]–[13] and point to an intriguing asymmetry in the development of children’s aversion to disadvantageous and advantageous inequity. Recent studies have found that children as young as 3 years of age develop an aversion to disadvantageous inequity [14]–[17] but do not develop an aversion to advantageous inequity until later, around 8 years of age [14], [18]. In addition to developmental studies, experiments on nonhuman animals have raised the question of whether inequity aversion is unique to humans and have demonstrated that some nonhuman animals are sensitive to disadvantageous resource distributions [19]–[30]. These studies suggest that an aversion to disadvantageous inequity may have deep evolutionary roots. As yet, however, no study has directly tested advantageous inequity aversion in nonhumans and thus there is currently no evidence that nonhuman animals are averse to advantageously unequal allocations (see Brosnan et al., 2010 [30] for an indirect test of advantageous inequity aversion in chimpanzees, *Pan troglodytes*). Together, results from studies of children and nonhuman animals suggest that separate evolutionary and developmental mechanisms underlie the two forms of inequity aversion.

Empirical demonstrations of inequity aversion across adults, children and nonhuman animals raise the question of how inequity aversion could have evolved, given that it motivates individuals to sacrifice personal gain. Theories to explain the evolution and expression of inequity aversion can be broadly grouped under two hypotheses. First, the *Social Hypothesis* [4], [31]–[32] suggests that inequity aversion is specific to the social domain and evolved as a means of regulating contributions to, and payoffs from, cooperative interactions. According to this hypothesis an aversion to inequity allows individuals to ensure that they are not contributing more or less to cooperative activities than fellow cooperators and thus protects individuals from being exploited and from exploiting others. Second, the *Nonsocial Hypothesis* suggests that inequity aversion is a result of domain-general mechanisms such as reference dependence and loss aversion that allows individuals to gauge their own payoffs relative to expected payoffs [33]–[35]. According to the Nonsocial Hypothesis, inequity aversion may *operate in* social interactions but did not necessarily evolve *for* social interactions per se. Sensitivity to lower-than-expected payoffs may indeed be useful even in non-cooperative contexts. For example, an attention to how one’s payoffs compare to available payoffs, including those of conspecifics, could confer a benefit in a foraging context where individuals can alter foraging strategies based on information about what payoffs can be expected in a given environment [33].

The Social and Nonsocial hypotheses generate different predictions. First, according to the Social Hypothesis, rejections of unequal allocations should occur only when resources are divided between social partners. Furthermore, individuals should only reject unequal allocations when their rejections affect their partner’s payoff and not when their partner’s payoff is fixed relative to their own. According to the Nonsocial Hypothesis, rejections of unequal allocations can occur even when there is no social partner. However, they should occur only in disadvantageous situations (i.e. small rewards will be less desirable when a larger possible reward is present for comparison) and not in advantageous situations where one’s payoff is already better than other available payoffs.

Distinguishing these hypotheses is critical to determining why humans show inequity aversion and to understanding the relationship between inequity aversion and fairness. Additionally, testing nonsocial influences on inequity aversion can shed light on the processes supporting the human aversion to disadvantageous and advantageous inequality. If disadvantageous inequity aversion is specifically social, then it is most likely linked to fairness concerns (i.e., it is not fair that I have less than someone else) and may thus have evolved for cooperation. However, if disadvantageous inequity aversion is a nonsocial response then it may not be tightly linked to fairness and may instead be related to maximizing personal rewards relative to available rewards. By contrast, advantageous inequity aversion should be specifically social and, as such, may represent a strong concern for fairness.

Only one study of inequity aversion in humans has directly compared a social with a nonsocial condition in a human allocation game. Sanfey et al. [36] found that rejections in the ultimatum game were higher when disadvantageous unequal offers were made by a human partner compared to a nonsocial condition where similar ‘offers’ were made by a computer. Notably, however, individuals also rejected many unequal offers made by the computer, even though no human partner would have received the better deal if the offer had been accepted. Thus, rejections of inequitable offers were stronger in a social context, suggesting that social influences play an important role in the expression of inequity aversion in human adults. However, results from Sanfey et al [36] demonstrate that inequity aversion in human adults is not necessarily restricted to situations where participants are interacting with a partner.

In contrast to studies of human adults, studies of inequity aversion in nonhuman animals have carefully examined the degree to which inequity aversion is specific to the social domain. Indeed, this issue has been discussed extensively because it is essential for the broader question of whether nonhuman primates demonstrate inequity aversion and, if so, whether animal inequity aversion is comparable to that of humans [19], [25], [31]–[32], [37]. One frequently cited experiment provides a useful example that is representative of the majority of animal inequity aversion tasks. In the first study of inequity aversion in a nonhuman species, Brosnan and de Waal [19] gave pairs of female capuchin monkeys (*Cebus apella*) equal payoffs or unequal payoffs in return for trading a token. Results showed that participants were least likely to trade a token when their partner received a high value reward for free while they had to trade a token for a low value food item. However, participants also showed high refusals in a nonsocial condition, where high value food was placed in an adjacent cage and they were given the option to trade for a low value item. The fact that participants refused trading opportunities in a nonsocial condition showed that while inequity aversion might be moderated by social context, it was not specific to the social context. Furthermore, offers were produced by a third party (i.e. the experimenter) and rejections did not actually affect the social partner’s payoff [37]. Given this, participants may have used rejections to elicit more favorable distributions from the experimenter.

As illustrated in the example above, Brosnan and de Waal’s [19] study and several similar nonhuman animal studies of inequity aversion have failed to provide strong support for the Social Hypothesis for two reasons. First, rejections of unequal offers are found regularly in nonsocial contexts [19]–[21], [24]–[26]. Second, animal tasks are typically designed such that recipients receive their payoffs regardless of the deciders’ decision [19]–[27], [37]. Thus, it is unclear why deciders would reject unequal offers given that, unlike human studies of inequity aversion, rejections do not affect the overall payoff distribution. One possibility is that rejections are simply a means of influencing the distributer (i.e. the experimenter) that participants desire a better reward.

Results from nonhuman animal studies raise important methodological concerns for the study of inequity aversion in humans. Manipulations of the social context and of the role of the experimenter are essential for understanding the mechanisms that underlie rejections of personal gain in reaction to inequity. Indeed, manipulations of this kind are critical to testing the Social and Nonsocial hypotheses for the evolution of inequity aversion.

Taken together, results from animal inequity aversion studies and from Sanfey et al (2003) [36] suggest that nonsocial factors may influence the expression of disadvantageous inequity aversion in humans and nonhuman species. What is currently unknown, however, is the extent to which the nonsocial dimension of inequity aversion is present in childhood. Furthermore, to understand whether social context differentially affects the expression of aversion to disadvantageous and advantageous inequity, it is essential to investigate the role of social influences on inequity aversion in a situation where these two processes are separable. Accordingly, we studied the role of social influences in the development of disadvantageous and advantageous inequity aversion in children, where an aversion to these two types of inequity follow different development trajectories.

To examine social influences on inequity aversion, we used a previously validated task: the Inequity Game [14]. The Inequity Game is a face-to-face task in which children are partnered with an unfamiliar peer. One child (the decider) decides whether to accept or reject allocations of candy, which are distributed by an experimenter. The decider’s decisions determine both their own and their partner’s payoffs. If a decider accepts an allocation, both children receive their respective payoffs. If a decider rejects an allocation, neither child receives any rewards.

The current study consists of two experiments. Experiment 1 asks whether children reject unequal reward allocations in an effort to solicit more favorable allocations from the experimenter. According to the Social Hypothesis, children reject inequity in order to deprive a partner of advantageous or disadvantageous payoffs. This assumes that the main social interaction in the Inequity Game is between the decider and his or her partner. Alternatively, the main social interaction in the Inequity Game may be independent of the partner’s presence and may instead be between the decider and the experimenter. In this scenario, rejections of unequal allocations may be an attempt to influence the experimenter’s allocation decisions. If this is the case, deciders should reject unequal allocations more frequently when the experimenter deliberately generates inequitable divisions of resources compared to when inequality is randomly generated. On the other hand, if children’s rejections are not intended to influence the experimenter, their frequency should not be affected by whether offers are made deliberately or randomly.

Experiment 2 provides a direct test of the Social Hypothesis by testing children using a nonsocial variation of the Inequity Game in which there is no recipient. If inequity aversion in children is a specifically social phenomenon, we expect few, if any, rejections in the nonsocial version of the game regardless of whether it involves advantageous or disadvantageous inequity. However, if the Nonsocial Hypothesis is true, children should continue to reject disadvantageous allocations in the same pattern as they did in the original, social version of the Inequity Game.

## General Method

### Inequity Game

**Figure 1:**
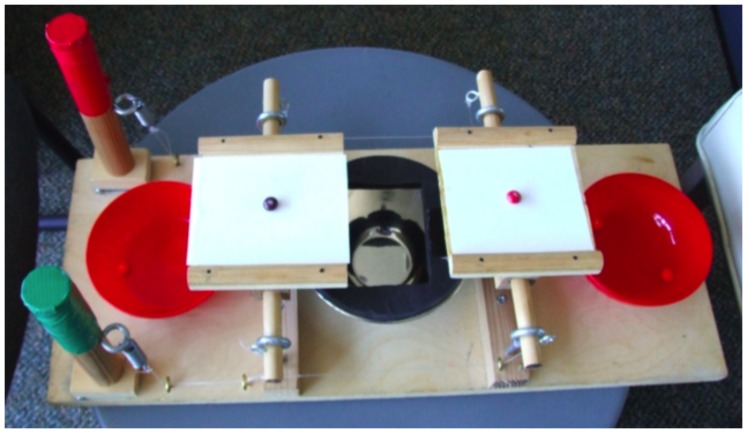
Photograph of apparatus used in these studies. Deciders sat on the left side of the apparatus and could operate the handles while the partner (if present) sat on the right side of the apparatus. Pulling the green handle caused the trays to tip outwards, delivering candies to the two outside bowls (“accepting an offer”). Pulling the red handle caused the trays to tip inwards, delivering candy to the inside bowl (“rejecting an offer”).

The method used in these studies closely follows that described in Blake and McAuliffe, 2011 [14]. In the original Inequity Game two children sat face-to-face and were assigned one of two roles. One child (“decider”) controlled a pair of handles, which were used to make decisions, while the other child (the “partner” or “recipient”) sat across from the decider and could not reach the handles. The experimenter placed allocations of Skittles® on both sides of the apparatus (Fig. 1), always placing the candies on the recipient’s side first in order to ensure that the decider paid attention to the recipient’s payoff before perceiving their own.

Before starting the game the experimenter demonstrated how the handles work: the decider could accept the allocation by pulling the green handle which tilted the trays outwards, causing Skittles to fall into bowls on each side of the apparatus. The decider could reject the allocation by pulling the red handle, which caused the trays to tip inwards, causing Skittles to fall into the middle bowl, where neither child was able to obtain them. Participants were told that any Skittles that fell into their bowls could be taken home at the end of the game but that neither they nor their partner would take home the Skittles in the middle bowl. Children were asked to move Skittles into two side bowls, located beside the apparatus, so that they could track the candies accumulating in each other’s bowls. Each side bowl was clearly associated with one of the participants. After the game was explained in this way, the participants were given practice trials to ensure that they understood the apparatus, including the effects of pulling both handles. The practice trials were as follows: 1–1 (one for decider, one for recipient); 0–1 (disadvantageous inequity; none for decider, one for recipient) and 1–0 (advantageous inequity; one for decider, none for recipient). If a participant accepted all warm-up trials, they were given an extra 1–1 trial and asked to try the red handle. Children were not instructed to stay silent during the game. Participants’ parents were in the vicinity of the testing area and could watch the game but could not interfere (sessions were excluded in the case of parental interference, see below).

### Design

Participants for Experiments 1 and 2 were recruited in public parks around Boston between July 2009 and August 2010. Participants were pseudo-randomly assigned to experiment.

### Analyses

All statistical analyses were conducted with R statistical software (version 2.15.2) [38]. Decision data were analyzed using Generalized Linear Mixed Models (GLMMs) with a binary response term (accept or reject) [39]. Mixed models were run using the package ‘lme4’ [40]. In all models participant identity (ID) was fit as a random effect to control for repeated measures.

Our GLMM procedure was as follows: (1) we examined a null model, which included participant ID as the only explanatory variable to test how much variation in the response term could be accounted for by individual variation; (2) we created a full model, which included predictor variables and all two-way interactions between Distribution (equal vs. unequal) and the other predictor variables (see Table 1 for a description of predictor variables); (3) the full model was compared to the null model using a likelihood ratio test (LRT) to test whether the inclusion of predictors provided a better fit to the data than participant ID alone. Unless otherwise noted, full models provided a better fit to data than null models; (4) a minimal model was created from the full model by sequentially dropping single terms from the model and testing whether their inclusion improved the model fit using likelihood ratio tests.

**Table 1:** Description of predictor variables used in analyses of children’s decisions to accept or reject reward allocations in Experiment 1 and Experiment 2.

Condition	Fixed effect with two levels: disadvantageous inequity, advantageous inequity
Distribution	Fixed effect with two levels: equal (1–1), unequal (disadvantageous inequity: 1–4 or advantageous inequity: 4–1)
Age group	Fixed effect with three levels: 4&5, 6&7, 8&9
Decider gender	Fixed effect with two levels: male, female
Origin^1^	Fixed effect with two levels: deliberate, random
Order^1^	Fixed effect with two levels: deliberate block first, random block first
Order^2^	Fixed effect with two levels: equal block first, unequal block first

1 Variable is unique to Experiment 1.

2 Variable is unique to Experiment 2.

To examine whether children’s decision varied over test trials, we used Wilcoxon signed-rank tests. Results from trial analyses were not significant unless reported. All tests were two-tailed and alpha was set at 0.05. Figures show raw data and were created using the ‘ggplot2’ package [41]. Binomial confidence intervals were calculated using the Agresti-Coull method [42].

### Ethics

This study was approved by Harvard University’s Committee on the Use of Human Subjects in Research. Guardians of participants gave informed consent in writing before children participated in the study.

Experiment 1: Are Children Attempting To Influence The Experimenter?

We tested whether children were more likely to accept unfair offers that were not under the experimenter’s control compared to those that were under the experimenter’s control. To this end, we performed the Inequity Game with a decider and a partner sitting face-to-face and we manipulated the origin of the offers such that half of the trial distributions were deliberately determined by the experimenter (hereafter, “deliberate” offers) while the other half of trial distributions were randomly determined by cards (hereafter, “random” offers) that had different distributions printed on them (see Fig. S1 for an illustration of cards).

### Methods

**Participants.** Children aged 4–9 were recruited in public parks in the Boston area. Parents were approached and asked if their child would be interested in participating in a game where she/he gets to take home candy. If parents consented, children were escorted to a testing area containing the Inequity Game test apparatus. We tested a total of 124 pairs (decider age range 4;0–9;9, 59 female deciders). Participant information for Experiment 1 is reported in Table S1. An additional 16 participants were tested but excluded due to experimenter error (13), parental interference (2) or discomfort (1).

**Design.** Children were assigned to one of two conditions: disadvantageous inequity (N = 64, 26 female deciders) or advantageous inequity (N = 60, 33 female deciders). Allocation origin (deliberate or random) and distribution (equal or unequal) were tested within participants, and inequity type (advantageous or disadvantageous) was a between-subject factor. This meant that each pair of children received three deliberate equal allocations (1–1), three deliberate unequal allocations (either disadvantageous, 1–4, or, advantageous, 4–1), three random equal allocations (1–1) and three random unequal allocations (either disadvantageous, 1–4, or, advantageous, 4–1). Allocation origin was blocked so that pairs received six random allocations followed by six deliberate allocations or vice versa, with equal and unequal trials randomized within block.

**Procedure.** Before administering the randomly generated allocations, the experimenter showed the participants the cards and explained how they determined the distribution. The decider was then asked two questions to make sure she/he understood that the allocations were not under the experimenter’s control. First, the experimenter asked the child “*Do you know what the next card will be?*” and then “*Do I know what the next card will be*?” If a participant did not say “no” to these two questions, the experimenter stated that the distribution would be a surprise for everyone. The majority of children spontaneously answered these questions correctly. However, 24 children did not (17 children in disadvantageous inequity; 7 children in advantageous; 19% of total sample). The pattern of our results held regardless of whether these children were included in analyses (see Table S5 and Fig. S4). On each random allocation trial, the experimenter revealed the card to the child and distributed Skittles in accordance with the depicted allocation.

If parents consented, we videotaped sessions (93% of sessions). Data were analyzed from video coding for these sessions (115 out of 124) and from live coding for the non-recorded sessions (9 sessions).

### Results

**Figure 2:**
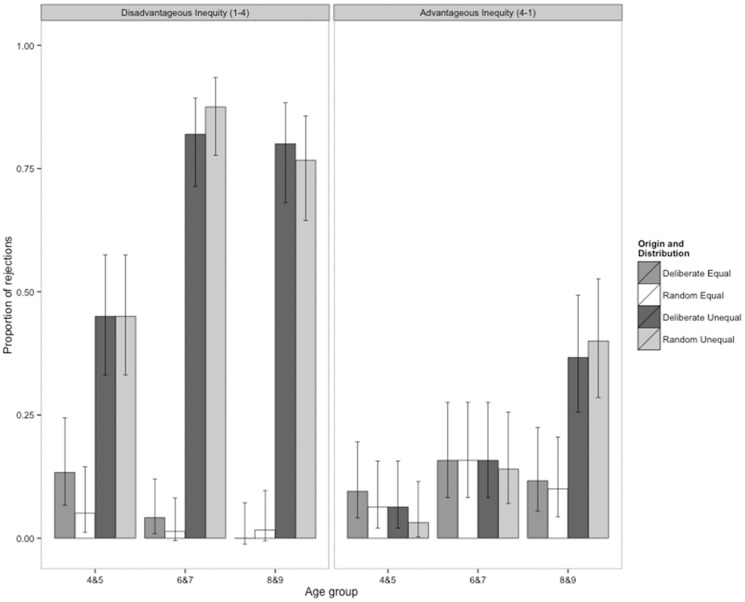
Proportion of reward allocations rejected in Experiment 1, in which reward allocations were either generated deliberately by the experimenter or randomly generated by a deck of cards. Rejections are shown for the disadvantageous inequity condition (A) and the advantageous inequity condition (B). Participants were assigned either to the disadvantageous inequity condition (*N*  =  64 pairs) or to the advantageous inequity condition (*N*  =  60 pairs). In the disadvantageous inequity condition, participants received one piece of candy while either one piece (equal distribution) or four pieces (unequal distribution) were placed on the recipient’s side of the apparatus. In the advantageous inequity condition, participants received either one piece of candy (equal distribution) or four pieces (unequal distribution) while one piece was placed on the recipient’s side of the apparatus. In both the disadvantageous inequity and advantageous inequity conditions, participants received three of each trial type: 1) deliberate equal; 2) random equal; 3) deliberate unequal and 4) random unequal. Error bars represent 95% confidence intervals.

Results from Experiment 1 are shown in Fig. 2a and 2b. This figure illustrates that children responded differently to the two types of inequality, rejecting more allocations in the disadvantageous inequity condition than in the advantageous inequity condition. In contrast, their rejections of equal allocations were similar across both conditions. This observed interaction between Distribution (equal vs. unequal) and Condition (disadvantageous inequity vs. advantageous inequity) was a significant predictor of children’s decisions in our minimal model (LRT, *X* ^2^_1_  =  123.97, *P* < 0.001). Because participants’ decisions about reward allocations differed between conditions, all subsequent analyses were conducted separately for disadvantageous and advantageous inequity.

Results from the disadvantageous inequity condition are shown in Fig. 2a. The main question motivating our analysis was whether children were more likely to reject disadvantageous, unequal allocations that were deliberately, as opposed to randomly, generated. As Fig.2a shows, children did not distinguish between these two allocation origins. A full GLMM of children’s decisions in the disadvantageous inequity condition showed that the interaction between Origin and Distribution was not significant (LRT, *X* ^2^_1_  =  2.45, *P*  =  0.118). We thus dropped this interaction from the model when creating the minimal model and additionally asked whether there was a main effect of Origin. This factor was not a significant predictor of children’s decisions (LRT, *X* ^2^_1_  =  0.23, *P*  =  0.635). Given that the origin of disadvantageous inequity allocations did not affect children’s decisions, we eliminated both the Origin and Order (deliberate or random block first) terms from our model.

Our minimal model (see Table S2 for model output) showed that there were two significant predictors of participants’ decisions in the disadvantageous inequity condition: (1) an interaction between Distribution and Age group (LRT, *X* ^2^_2_  =  35.19, *P* < 0.001) and (2) an interaction between Distribution and Decider gender (LRT, *X* ^2^_1_  =  5.61, *P*  =  0.018). Fig. 2a illustrates the interaction between Distribution and Age group: older children were more likely to reject unequal allocations than younger children but rejections of equal offers did not vary with age. The interaction between Decider gender and Distribution was due to the fact that males were slightly more likely to reject equal offers and slightly less likely to reject unequal offers than girls in the disadvantageous inequity condition (see Fig. S2 for a depiction of this interaction).

We examined participants’ decisions in the advantageous inequity condition following the same steps as outlined above. As shown in Fig. 2b, children did not distinguish between deliberately generated allocations and randomly generated allocations. Indeed, GLMMs revealed that neither the interaction between Origin and Distribution nor the main effect of Origin were significant predictors of participants’ decisions in the advantageous inequity condition (*X* ^2^_1_  =  0.09, *P*  =  0.766, *X* ^2^_1_  =  0.22, *P*  =  0.638, respectively). Results from our minimal model showed that the only significant predictor of participants’ decisions in the advantageous inequity condition was the interaction between Distribution and Age Group (LRT, *X* ^2^_2_  =  20.77, *P* < 0.001; model output is shown in Table S2). Children across the three age groups were unlikely to reject equal offers and 4&5- and 6&7-year-olds rarely rejected advantageously unequal offers (see Fig. 2b). However, 8&9-year-olds tended to reject more unequal reward allocations than equal allocations.

### Discussion

We found that children’s levels of rejections did not differ between unequal allocations that were deliberately generated by the experimenter and allocations that were randomly generated by cards. Regardless of whether the distribution of rewards was randomly determined or chosen by the experimenter, 4- to 9-year-old children were likely to reject disadvantageous allocations. This suggests that children did not reject disadvantageous inequity in order to elicit more favorable distributions from the experimenter. Similarly, children in the 8&9-year-old age group rejected more advantageous allocations than equal allocations, irrespective of whether the experimenter had control over allocations. This result is congruent with Blake and McAuliffe (2011) [14] in showing that advantageous inequity aversion emerges at 8–9 years. Further, our findings importantly extend previous work by showing that rejections of advantageous allocations are a response to an unequal resource distribution between two peers and are not an attempt to influence the experimenter.

It is possible that children may not have understood the card manipulation and instead assumed that the experimenter was in control regardless of how allocations were determined. This seems unlikely because the majority of children (81%) answered our card comprehension questions correctly, confirming that they understood that the experimenter did not know what the next allocation would be. Moreover, the pattern of our results held even when participants who did not correctly answer comprehension questions were excluded from analyses. Furthermore, previous work shows that children between 4 and 9 years of age distinguish intentional from accidental outcomes and have a basic understanding of randomness [43]–[44]. Therefore, the most plausible interpretation of our results appears to be that children’s choices were guided by the allocations themselves and not by knowledge of whether allocations had been determined by the experimenter or not.

Findings from Experiment 1 suggest that the main social interaction in the Inequity Game is between the decider and the recipient as opposed to between the decider and the experimenter. This finding is also consistent with the idea that children reject reward allocations in order to prevent their partner from receiving a more desirable allocation (disadvantageous inequity) or a less desirable allocation (advantageous inequity). However, an alternative explanation for rejections in the Inequity Game is that children are opposed to the unequal reward allocations themselves. In other words, it is possible that children would reject unequal allocations regardless of whether or not they were paired with a social partner.

Understanding whether children are responding to the unequal allocations themselves or to an unequal division of rewards between themselves and a partner will help distinguish between the Social and Nonsocial hypotheses for the expression of inequity aversion. If children do indeed respond to the unequal allocations themselves, which is an alternative explanation for disadvantageous, but not advantageous inequity aversion, this result would be consistent with the Nonsocial Hypothesis. To address this alternative explanation for rejections of inequity, we conducted a nonsocial version of the Inequity Game in which children were faced with unequal outcomes in the absence of a social partner.

### Experiment 2: Do Children Reject Inequity in a Nonsocial Game?

The goal of this experiment was to test whether children’s rejections of unequal allocations in the Inequity Game are specific to situations in which deciders are paired with a social partner. To this end, we conducted the Inequity Game with a decider but no recipient. We reasoned that if children reject allocations due to an aversion to the unequal outcomes themselves, then rates of rejection in Experiment 2 should be indistinguishable from those observed in Experiment 1. However, if children are importantly influenced by the presence of a social partner, we should expect to see a difference in rates of rejections between the two studies.

### Methods

**Participants and design.** We tested a total of 201 children (107 females). As in Experiment 1, children were assigned to one of two conditions: disadvantageous inequity (N  =  98, 55 females; age range: 4;0–9;9); and advantageous inequity, N  =  103, 52 females; age range: 4;0–9;8). Participant information for Experiment 2 is reported in Table S1. An additional five participants were tested but excluded due to experimenter error (2), session interruption (1), parental interference (1) or shyness (1).

Children were given 3 warm-up trials and 12 test trials. Children participated in either the disadvantageous inequity condition or the advantageous inequity condition (between-subject factor). In both conditions, the test trials were blocked so that children received a block of 6 equal trials (1–1, 1 for decider, 1 on the other tray) and a block of 6 unequal trials (disadvantageous inequity: 1 for decider, 4 on other tray; advantageous inequity: 4 for decider, 1 on the other tray). Block order was counterbalanced across participants.

**Procedure.** Children were recruited in public parks, as described in Experiment 1. The instructions were the same as above except that, here, the experimenter said that the Skittles on the other side of the apparatus would go back into the bag at the end of the game. To test their understanding of this, children were asked where the Skittles on the other side of the apparatus would go at the end of the game. If children failed to spontaneously answer this question correctly (15 children; 7 children in disadvantageous inequity and 8 in advantageous inequity; 7.5% of total sample), the experimenter would restate that the Skittles went back in the bag at the end of the game. Excluding children who did not answer this question correctly did not change the pattern of our results.

Video recordings were available for 98.5% of participants and unavailable for three participants for whom we did not have video consent. Data were analyzed from video coding for all but these sessions. Data from live coding were analyzed for the three non-recorded sessions.

### Results

**Figure 3:**
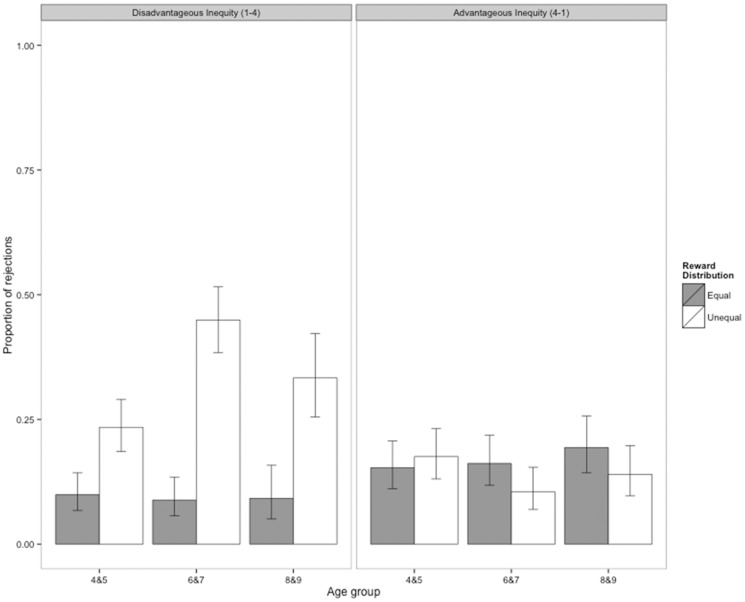
Proportion of reward allocations rejected in Experiment 2, the nonsocial version of the Inequity Game. Rejections are shown for the disadvantageous inequity condition (A) and the advantageous inequity condition (B). Participants were assigned either to the disadvantageous inequity condition (*N*  =  98) or to the advantageous inequity condition (*N*  =  103). In the disadvantageous inequity condition, participants received one piece of candy while either one piece (equal distribution) or four pieces (unequal distribution) were placed on the other side of the apparatus. In the advantageous inequity condition, participants received either one piece of candy (equal distribution) or four pieces (unequal distribution) while one piece was placed on the other side of the apparatus. In both the disadvantageous inequity and advantageous inequity conditions, participants received six equal and six unequal trials. Error bars represent 95% confidence intervals.

**Nonsocial Game.** Results from Experiment 2 are shown in Fig. 3a and b. Children responded differently to the two types of inequality, rejecting more unequal distributions in the disadvantageous inequity condition than in the advantageous inequity condition. By contrast, their rejections of equal distributions were similar across both conditions. As in Experiment 1, we found that the interaction between Condition (disadvantageous vs. advantageous inequity) and Distribution (equal vs. unequal) was a significant predictor of children’s decisions (LRT, *X* ^2^_1_  =  74.91, *P* < 0.001). Consequently, all subsequent analyses were conducted separately for disadvantageous and advantageous inequity conditions.

Fig. 3a illustrates children’s probability of rejecting unequal compared to equal allocations in the disadvantageous inequity condition. Examination of this figure suggests that children in all age groups rejected more unequal offers (1–4) than equal offers (1–1). Furthermore, this figure indicates that older children were more likely to reject unequal offers than younger children. In contrast, rejections of equal offers were low overall, and stable across age groups. Indeed, our minimal model confirmed that interaction between Age Group and Distribution was a significant predictor of children’s decisions in the disadvantageous inequity condition (LRT, *X* ^2^_2_  =  10.03, *P*  =  0.007; see Table S3 for model output).

Results for the advantageous inequity condition are shown in Fig. 3b. As this figure illustrates, children rarely rejected unequal offers that benefited them more (4–1). Indeed, neither Age Group nor Distribution predicted rejections in our game. Our GLMM analyses showed that a full model, including all predictors and two-way interactions with Distribution, provided only a marginally better fit to participants’ decision data than a null model that included participant ID as the sole explanatory term (*X* ^2^_9_  =  16.51, *P*  =  0.057). This finding suggests that inter-individual variation accounted for almost as much variation in participant behavior as did predictor variables and participant ID combined.

Our minimal model showed that the only significant predictor of children’s behavior was the order in which blocks of trials were presented (LRT, *X* ^2^_1_  =  7.50, *P*  =  0.006; see Table S3 for model output). This order effect was due to the fact that children who received the 4–1 block first rejected more trials overall (mean rejections overall  =  1.2, mean rejections of 1–1  =  1.4, mean rejections of 4–1  =  1.0) compared to children who received the 1–1 block first (mean rejections overall  = .65, mean rejections of 1–1  = .66, mean rejections of 4–1  = .64).

We were interested in whether children’s decisions varied across trials. To test this, we performed Wilcoxon signed-rank tests on participants’ first three unequal trials compared to their last three unequal trials. We also examined whether participants’ decisions about equal trials varied across trials using these same comparisons. Separate Wilcoxon signed-rank tests were performed for each age group within each condition (see Fig. S3 for a graph showing decisions over trials). In two cases, we found a significant difference between the first and second block of three unequal trials. Children in the 6&7-year-old age group were less likely to reject disadvantageously unequal trials in the second group of three trials compared to the first group of three trials (*W*  =  833, *P*  =  0.030). Similarly, children in the 8&9-year-old age group were less likely to reject disadvantageously unequal allocations in later trials (*W*  =  269.5, *P*  =  0.049). None of the other comparisons showed a significant difference between the first three and second three trials (*P*s > 0.2).

**Figure 4:**
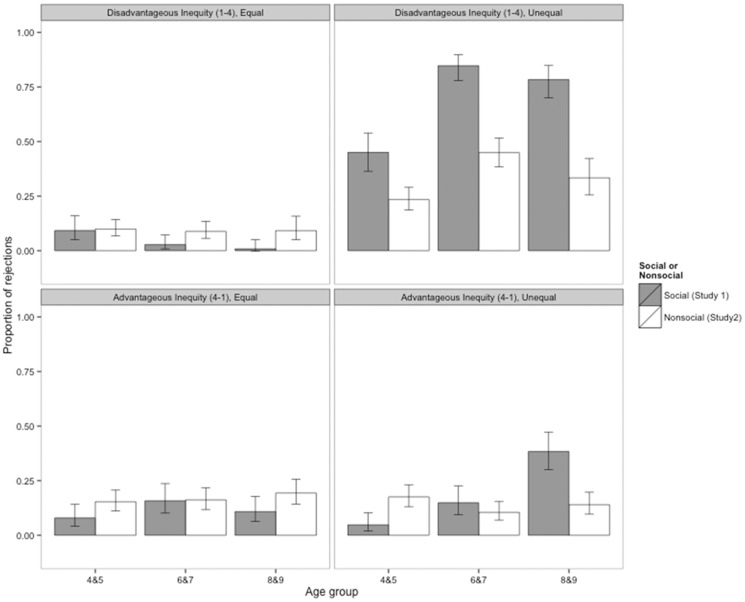
Proportions of reward allocations rejected in Experiments 1 (social) and 2 (nonsocial). Rejections are shown for the disadvantageous inequity condition (A and B) and the advantageous inequity condition (C and D). Within condition, rejections are shown by equal distribution (1–1, A and C) and unequal distribution (1–4 of 4–1, B and D). Participants were assigned either to the disadvantageous inequity condition or to the advantageous inequity condition. Within condition, participants received six equal trials and six unequal trials. Error bars represent 95% confidence intervals.

**Experiment 1 and Experiment 2 compared.** To examine whether children rejected more disadvantageous inequity and advantageous inequity offers in the social version of the game (i.e., when they were paired with a partner) than the nonsocial game, we compared results from Experiments 1 and 2. Figure 4a-d illustrate children’s probability of rejection in the social and nonsocial versions of the Inequity Game. Children’s rejections are shown separately by condition and distribution to reflect our method of analysis.

To address the question of whether rejections varied by social context (i.e. Experiment 1 or Experiment 2), we conducted four separate GLMMs that each tested whether participants’ decisions were predicted by an interaction between Experiment (social, i.e. Experiment 1 or nonsocial, i.e. Experiment 2) and Age group. For the equal allocations, results from these models showed that children’s rejections did not depend on social context (see Table S4 for model output). The interaction between Age group and Experiment was not significant for either the disadvantageous inequity or advantageous inequity condition (disadvantageous inequity: *X* ^2^_2_  =  4.05, *P*  =  0.132; advantageous inequity: *X* ^2^_2_  =  1.14, *P*  =  0.566).

In contrast, for the unequal reward allocations, children’s decisions did vary by experiment. The interaction between Age group and Experiment was a significant predictor of children’s decision in both the disadvantageous inequity and advantageous inequity conditions (disadvantageous inequity: *X* ^2^_2_  =  30.03, *P* < 0.001; advantageous inequity: *X* ^2^_2_  =  7.26, *P*  =  0.027). Figure 4b and 4d illustrate these interactions. In the disadvantageous inequity condition, children in all age groups rejected unequal allocations more often in the social than the nonsocial version of the Inequity Game. In the advantageous inequity condition, 8&9-year-old children rejected unequal offers (4–1) more often in the social game than in the nonsocial game. However, 4&5- and 6&-7-year-olds’ rejections of unequal reward allocations in the advantageous inequity condition did not differ between social and nonsocial contexts.

### Discussion

There are three major findings from Experiment 2. First, 4- to 9-year-old children tended to reject disadvantageous inequity allocations in a nonsocial situation. To our knowledge, this is the first study to demonstrate that children will reject inequity in a nonsocial version of a reward distribution game. Second, 4- to 9-year-old children tended to reject disadvantageous inequity significantly more often when they were playing with a social partner than when they were playing the nonsocial game. Third, whereas younger children accepted advantageous inequity allocations in both the nonsocial and the social versions of the game, 8&9-year-old children rejected advantageous allocations only when they were paired with a social partner.

The fact that children in a nonsocial game often rejected disadvantageous inequity allocations suggests that their rejections in the social version of this game were not motivated purely by an aversion to having a smaller payoff than a social partner (i.e., envy). Rather, in both nonsocial and social contexts, children may have rejected disadvantageous inequity allocations in part because their payoff was relatively less than other potential payoffs. Rejections of disadvantageous inequity allocations in a nonsocial context are thus consistent with the Nonsocial Hypothesis that inequity aversion is built on a heuristic for gauging the relative value of one’s payoff compared to an expected payoff (e.g. reference-dependence) [33]–[35], [45]. In the disadvantageous inequity condition, children may have been comparing their allocations of Skittles to other available allocations (i.e. they are comparing their single skittle to the possible allocation of four Skittles) regardless of whether another individual was benefiting from the differential payoff distribution. However, this reference-dependence explanation cannot fully account for children’s rejections in the social game because rejections were significantly higher there than in the nonsocial version of the game. Thus, nonsocial influences partially explain disadvantageous inequity aversion in children, but the presence of a social partner increases children’s aversion to disadvantageous reward distributions.

In contrast to the disadvantageous condition, results from the advantageous inequity condition show that children only rejected advantageous allocations when playing the social version of the task: they accepted advantageous inequity allocations in the nonsocial task. This highlights that advantageous inequity aversion is a genuinely social phenomenon and cannot be explained by nonsocial reference-dependence. Moreover, this finding provides further evidence for the notion that disadvantageous inequity and advantageous inequity aversion follow different developmental pathways and hence may be underpinned by different psychological mechanisms.

## General Discussion

Combined, these two experiments provide a detailed picture of how social influences affect children’s decisions about unequal payoffs. Experiment 1 demonstrated that children were not using rejections as a means of eliciting more favorable distributions from the experimenter and, thus, that the main social interaction in the Inequity Game was between the decider and their social partner. Experiment 2 showed that social partners influenced how children reacted to inequity, although their importance varied depending on the form of inequity. An aversion to advantageous inequity is clearly a specifically social phenomenon; 8&9-year-old children only rejected advantageous inequity when a partner was present. Disadvantageous inequity aversion, on the other hand, has an important nonsocial component; children in all age groups rejected some disadvantageous inequity allocations in the absence of a social partner. Importantly, however, disadvantageous inequity aversion is influenced by social context; children rejected more disadvantageous inequity allocations in the social game than in the nonsocial game.

In Experiment 1, the experimenter’s intentional delivery of unequal allocations had no effect on children’s decisions, suggesting that the main social interaction in the task was between decider and recipient rather than between the decider and experimenter. Moreover, this demonstrates that rejections in the Inequity Game were not attempts to influence the experimenter’s reward allocations but were based instead on the relative rewards at stake. Additionally, Experiment 1 provides an independent replication of the age-shift reported in Blake and McAuliffe [14] with 8&9-year-old children rejecting advantageous allocations when playing the Inequity Game with a social partner.

The results of Experiment 2 provided support for the idea that advantageous and disadvantageous inequity aversion are supported by two different cognitive processes [14], [18]. Specifically, 8&9-year-olds rejected advantageous offers only if there was a social partner who would get less than them; children at this age accepted advantageous offers in the nonsocial version. These results are consistent with the idea that advantageous inequity aversion evolved *for* social interactions and is not based on domain-general mechanisms.

Results from the disadvantageous inequity conditions, on the other hand, suggest that both social and nonsocial factors might contribute to disadvantageous inequity aversion. In Experiment 2, 4- to 9-year-old children rejected disadvantageous inequity allocations at significant levels even when no peer would receive the larger reward. The fact that children in the nonsocial game would rather have nothing than accept a relatively small reward suggests that disadvantageous inequity aversion in children has an important nonsocial component. This result is surprising in light of work on adults where it is generally assumed that inequity aversion is a specifically social phenomenon and, thus, nonsocial tests are not typically conducted (see Sanfey et al. [36] for an exception).

Although there are clearly important social influences on disadvantageous inequity aversion in children, disadvantageous inequity aversion does not appear to be triggered exclusively by interactions with a social partner. Rather, our results suggest that, unlike advantageous inequity aversion, disadvantageous inequity aversion may be built on a simpler domain-general process like reference-dependence [33]–[35], which is consistent with the Nonsocial Hypothesis for the evolution of inequity aversion. Future work is necessary to understand the specific mechanisms that underpin rejections of disadvantageous inequity allocations in a nonsocial task, but, minimally, we can conclude from our results that it may be necessary to revise the commonly held view that individuals only reject disadvantageous allocations in order to influence a partner’s payoff. Furthermore, our results suggest that envy alone cannot account for rejections of disadvantageously unequal allocations. More broadly, we argue that a productive area for future work would be (1) to understand why advantageous inequity aversion is specifically social while disadvantageous inequity aversion is not and (2) to develop a theory for the evolution of inequity aversion that can account for this important dissociation by integrating the Social and Nonsocial hypotheses. Such an approach will also be instrumental in creating ties between studies of inequity across human adults, children and nonhuman animals.

Rejections of unequal allocations in the nonsocial game represent an intriguing similarity with nonhuman animal work where individuals commonly reject inequitable allocations in nonsocial controls [19]–[21], [24]–[26]. While results from Experiment 2 cannot speak directly to the evolutionary origin of inequity aversion in humans, they suggest at least two plausible explanations. First, it is possible that inequity aversion is indeed a purely social phenomenon in humans and rejections in the absence of a social partner are a misapplication of this aversion. In line with this hypothesis, children in our sample may have acquired an expectation about equity in the social domain and have erroneously applied this expectation to the nonsocial task. Alternatively, inequity aversion in humans may be built on domain-general mechanisms that are shared with nonhuman species [34] and that is enhanced by social context. In line with this view, children perceive their payoff of one Skittle as less desirable when it is distributed alongside of a payoff of 4 Skittles compared to when it is alongside of a payoff of 1 Skittle. Children may react aversively to this payoff asymmetry regardless of whether it is benefiting a peer, but their reactions to inequity are strongest when a peer benefits from the asymmetry. At present, we are unable to distinguish between these alternatives but view them as fruitful areas for future inquiry.

Experiment 1 was designed to test whether the critical social interaction in the Inequity Game is between decider and experimenter or between decider and recipient. We tested this by asking whether children were rejecting unequal allocations in the Inequity Game in order to elicit more favorable distributions from the experimenter. Results from this study show that deciders did not distinguish between unequal allocations that were deliberately versus randomly generated by the experimenter, suggesting that children were most likely not attempting to influence the experimenter with rejections. Further evidence in support of the idea that children did not reject unequal allocations in order to influence the experimenter comes from the finding that there was a difference in levels of rejections in the nonsocial and social versions of the Inequity Game. If children’s rejections in the game were solely motivated by a desire to influence the experimenter, we would not expect to see this difference. Given these two lines of reasoning, we argue that the relevant social interaction in the Inequity Game is between decider and recipient and that children show high levels of rejection in the social version of the Inequity Game, most likely because they are attempting to affect their social partner’s payoff through rejections.

More broadly, the results from Experiment 1 have important methodological implications because they demonstrate that children’s behavior in the Inequity Game is not driven by their desire to influence the experimenter. Given that almost all studies of inequity aversion in children are done in the presence of an experimenter, this may help alleviate concerns about experimenter effects and substantiate the interpretation that children’s decisions in these tasks result from their interaction with a peer.

Social influences are undoubtedly important in the expression of inequity aversion in children, and this is especially true for advantageous inequity aversion. However, there are also important nonsocial factors at play, as was evidenced by children’s rejections of disadvantageous allocations in the nonsocial game. Thus, our results begin to paint a more nuanced picture of the emergence of inequity aversion in children. Understanding the social factors that influence the expression of inequity aversion is critical to understanding its evolution and development but, to date, few studies have tested these influences empirically. Examining the social factors that influence inequity aversion in children and adults will help unite human inequity aversion studies with inequity aversion studies in nonhuman animals and will help shed light on the evolutionary and developmental processes that shape inequity aversion in humans.

## Supporting Information

Figure S1 Picture of cards used in Experiment 1 to randomly generate offers.
(DOCX)

Figure S2 Line plots showing the interaction between decider gender and distribution in the disadvantageous inequity condition of Experiment 1.
(DOCX)

Figure S3 Probability of reward allocation rejection over trials in Experiment 2, the nonsocial version of the inequity game.
(DOCX)

Figure S4 Proportion of reward allocations rejected in Experiment 1 by participants who spontaneously answered the randomization comprehension questions correctly.
(DOCX)

Table S1 Number of children who participated in Experiments 1 and 2.
(DOCX)

Table S2 GLMM output: participants’ decisions in the disadvantageous and advantageous inequity conditions of Experiment 1.
(DOCX)

Table S3 GLMM output: participants’ decisions in the disadvantageous and advantageous inequity conditions of Experiment 2.
(DOCX)

Table S4 GLMM output: participants’ decisions in the disadvantageous inequity (DI) and advantageous inequity (AI) conditions of Experiments 1 and 2 combined.
(DOCX)

Table S5 GLMM output: decisions in the disadvantageous and advantageous inequity conditions of Experiment 1 for participants who spontaneously answered the randomization comprehension questions correctly.
(DOCX)
