# Supplementary material for: Social Influences on Inequity Aversion in Children
Source: PLoS One. 2013 Dec 2;8(12):e80966. doi: 10.1371/journal.pone.0080966 (PMC3846671; doi:10.1371/journal.pone.0080966)
Supplement: Figure S1 — Picture of cards used in Experiment 1 to randomly generate offers. (DOCX) [file pone.0080966.s001.docx]

**Figure S1**.

Picture of cards used in Experiment 1 to randomly generate offers. The black circle indicates the decider’s reward allocation. From left to right, cards show an equal allocation (1-1), a disadvantageous inequity allocation (1-4) and an advantageous inequity allocation (4-1).
