# Supplementary material for: Social Influences on Inequity Aversion in Children
Source: PLoS One. 2013 Dec 2;8(12):e80966. doi: 10.1371/journal.pone.0080966 (PMC3846671; doi:10.1371/journal.pone.0080966)
Supplement: Figure S2 — Line plots showing the interaction between decider gender and distribution in the disadvantageous inequity condition of Experiment 1. (DOCX) [file pone.0080966.s002.docx]

**Figure S2.**

Line plots showing the interaction between decider gender and distribution in the disadvantageous inequity condition of Experiment 1.
