# Supplementary material for: Social Influences on Inequity Aversion in Children
Source: PLoS One. 2013 Dec 2;8(12):e80966. doi: 10.1371/journal.pone.0080966 (PMC3846671; doi:10.1371/journal.pone.0080966)
Supplement: Figure S3 — Probability of reward allocation rejection over trials in Experiment 2, the nonsocial version of the inequity game. (DOCX) [file pone.0080966.s003.docx]

**Figure S3**.

Probability of reward allocation rejection over trials in Experiment 2, the nonsocial version of the inequity game. Rejections are shown across age groups for the disadvantageous inequity condition (top row) and the advantageous inequity condition (bottom row). Participants were assigned either to the disadvantageous inequity condition (*N* = 98) or to the advantageous inequity condition (*N* = 103). In the disadvantageous inequity condition, participants received one piece of candy while either one piece (equal distribution) or four pieces (unequal distribution) were placed on the other side of the apparatus. In the advantageous inequity condition, participants received either one piece of candy (equal distribution) or four pieces (unequal distribution) while one piece was placed on the other side of the apparatus. In both the disadvantageous inequity and advantageous inequity conditions, participants received six equal trials and six unequal trials.
