# Supplementary material for: Social Influences on Inequity Aversion in Children
Source: PLoS One. 2013 Dec 2;8(12):e80966. doi: 10.1371/journal.pone.0080966 (PMC3846671; doi:10.1371/journal.pone.0080966)
Supplement: Figure S4 — Proportion of reward allocations rejected in Experiment 1 by participants who spontaneously answered the randomization comprehension questions correctly. (DOCX) [file pone.0080966.s004.docx]

**Figure S4**.

Proportion of reward allocations rejected in Experiment 1 by participants who spontaneously answered the randomization comprehension questions correctly.
