# Supplementary material for: Social Influences on Inequity Aversion in Children
Source: PLoS One. 2013 Dec 2;8(12):e80966. doi: 10.1371/journal.pone.0080966 (PMC3846671; doi:10.1371/journal.pone.0080966)
Supplement: Table S1 — Number of children who participated in Experiments 1 and 2. (DOCX) [file pone.0080966.s005.docx]

**Table S1**. Number of children who participated in Experiments 1 and 2. Table shows numbers of deciders by Experiment, Condition (*DI* Disadvantageous Inequity, *AI* Advantageous Inequity), Age Group, and Decider Gender (*F* Female, *M* Male).

|  | | 4&5 | | 6&7 | | 8&9 | | Total |
| --- | --- | --- | --- | --- | --- | --- | --- | --- |
|  |  | F | M | F | M | F | M |  |
| Experiment 1:  Deliberate versus Random | DI | 7 | 13 | 10 | 14 | 9 | 11 | 64 |
|  | AI | 12 | 9 | 10 | 9 | 11 | 9 | 60 |
| Experiment 2:  Nonsocial | DI | 22 | 20 | 23 | 13 | 10 | 10 | 98 |
|  | AI | 19 | 18 | 13 | 22 | 20 | 11 | 103 |
