# Supplementary material for: Social Influences on Inequity Aversion in Children
Source: PLoS One. 2013 Dec 2;8(12):e80966. doi: 10.1371/journal.pone.0080966 (PMC3846671; doi:10.1371/journal.pone.0080966)
Supplement: Table S3 — GLMM output: participants’ decisions in the disadvantageous and advantageous inequity conditions of Experiment 2. (DOCX) [file pone.0080966.s007.docx]

**Table S3**. Output from minimal generalized linear mixed model of participants’ decisions in the disadvantageous and advantageous inequity conditions of Experiment 2. Coefficients indicate the estimated effects of predictors on the response term (accept = 1, reject = 0) relative to the following baseline levels: Distribution = equal; Age group = 4&5-year-old; Order = Equal block first.

| Experiment 2 |  |  | β | s.e. | z | p |
| --- | --- | --- | --- | --- | --- | --- |
| Disadvantageous Inequity |  | Intercept | 2.68 | 0.29 | 9.25 | <0.001 |
|  | Distribution | Unequal | -1.18 | 0.28 | -4.25 | <0.001 |
|  | Age group | 6&7-year-olds | 0.07 | 0.43 | 0.16 | 0.876 |
|  |  | 8&9-year-olds | -0.01 | 0.51 | -0.03 | 0.978 |
|  | Distribution x Age group | Unequal x 6&7-year-olds | -1.28 | 0.41 | -3.13 | 0.002 |
|  |  | Unequal x 8&9-year-olds | -0.63 | 0.48 | -1.3 | 0.193 |
| Advantageous Inequity |  | Intercept | 2.67 | 0.24 | 11.15 | <0.001 |
|  | Order | Unequal block first | -0.88 | 0.32 | -2.78 | 0.005 |
