# Supplementary material for: Social Influences on Inequity Aversion in Children
Source: PLoS One. 2013 Dec 2;8(12):e80966. doi: 10.1371/journal.pone.0080966 (PMC3846671; doi:10.1371/journal.pone.0080966)
Supplement: Table S4 — GLMM output: participants’ decisions in the disadvantageous inequity (DI) and advantageous inequity (AI) conditions of Experiments 1 and 2 combined. (DOCX) [file pone.0080966.s008.docx]

**Table S4**. Output from generalized linear mixed model of participants’ decisions in the disadvantageous inequity (DI) and advantageous inequity (AI) conditions. Separate models were run to examine participants’ decisions in a social context (Experiment 1) compared to a nonsocial context (Experiment 2). Models examined participants decisions about equal reward allocations (1-1) or unequal reward allocations (DI: 1-4; AI: 4-1) Coefficients indicate the estimated effects of predictors on the response term (accept = 1, reject = 0) relative to the following baseline levels: Social or nonsocial = nonsocial; Age group = 4&5-year-olds).

|  |  |  | β | s.e. | z | p |
| --- | --- | --- | --- | --- | --- | --- |
| DI: Unequal |  | Intercept | 1.63 | 0.29 | 5.59 | p < 0.001 |
|  | Social or nonsocial | Social | -1.91 | 0.50 | -3.82 | p < 0.001 |
|  | Age group | 6&7-year-olds | -1.37 | 0.42 | -3.28 | 0.001 |
|  |  | 8&9-year-olds | -0.69 | 0.50 | -1.39 | 0.166 |
|  | Social or nonsocial x Age group | Unequal x 6&7-year-olds | 4.03 | 0.72 | 5.58 | 0.000 |
|  |  | Unequal x 8&9-year-olds | 2.70 | 0.77 | 3.51 | 0.000 |
| DI: Equal |  | Intercept | 3.21 | 0.40 | 7.95 | p < 0.001 |
|  | Social or nonsocial | Social | -6.60 | 0.73 | -8.98 | p < 0.001 |
|  | Age group | 6&7-year-olds | 0.12 | 0.60 | 0.20 | 0.845 |
|  |  | 8&9-year-olds | 0.13 | 0.72 | 0.18 | 0.859 |
|  | Social or nonsocial x Age group | Unequal x 6&7-year-olds | -1.36 | 1.18 | -1.15 | 0.248 |
|  |  | Unequal x 8&9-year-olds | -2.62 | 1.84 | -1.43 | 0.154 |
| AI: Unequal |  | Intercept | 2.93 | 0.55 | 5.34 | p < 0.001 |
|  | Social or nonsocial | Social | -8.31 | 1.31 | -6.33 | p < 0.001 |
|  | Age group | 6&7-year-olds | 1.12 | 0.85 | 1.32 | 0.188 |
|  |  | 8&9-year-olds | 0.92 | 0.88 | 1.04 | 0.298 |
|  | Social or nonsocial x Age group | Unequal x 6&7-year-olds | 0.31 | 1.73 | 0.18 | 0.855 |
|  |  | Unequal x 8&9-year-olds | 3.38 | 1.63 | 2.07 | 0.039 |
| AI: Equal |  | Intercept | 2.26 | 0.32 | 6.99 | p < 0.001 |
|  | Social or nonsocial | Social | -5.44 | 0.61 | -8.88 | p < 0.001 |
|  | Age group | 6&7-year-olds | -0.05 | 0.46 | -0.10 | 0.920 |
|  |  | 8&9-year-olds | -0.29 | 0.47 | -0.62 | 0.535 |
|  | Social or nonsocial x Age group | Unequal x 6&7-year-olds | 0.87 | 0.84 | 1.04 | 0.298 |
|  |  | Unequal x 8&9-year-olds | 0.58 | 0.86 | 0.68 | 0.498 |
|  |  |  |  |  |  |  |
