# Supplementary material for: Social Influences on Inequity Aversion in Children
Source: PLoS One. 2013 Dec 2;8(12):e80966. doi: 10.1371/journal.pone.0080966 (PMC3846671; doi:10.1371/journal.pone.0080966)
Supplement: Table S5 — GLMM output: decisions in the disadvantageous and advantageous inequity conditions of Experiment 1 for participants who spontaneously answered the randomization comprehension questions correctly. (DOCX) [file pone.0080966.s009.docx]

**Table S5**. Output from minimal generalized linear mixed model of participants’ decisions in the disadvantageous and advantageous inequity conditions of Experiment 1. Table shows results for only those participants who spontaneously answered the randomization comprehension questions correctly. Coefficients indicate the estimated effects of predictors on the response term (accept = 1, reject = 0) relative to the following baseline levels: Distribution = equal; Age group = 4&5-year-old; Decider gender = female.

| Experiment 1 |  |  | β | s.e. | z | p |
| --- | --- | --- | --- | --- | --- | --- |
| Disadvantageous Inequity |  | Intercept | 4.86 | 1.12 | 4.35 | < 0.001 |
|  | Distribution | Unequal | -5.53 | 1.11 | -4.97 | < 0.001 |
|  | Age group | 6&7-year-olds | -0.09 | 1.28 | -0.07 | 0.943 |
|  |  | 8&9-year-olds | 0.78 | 1.64 | 0.47 | 0.636 |
|  | Decider gender | Male | -0.71 | 1.23 | -0.57 | 0.567 |
|  | Distribution x Age group | Unequal x 6&7-year-olds | -2.51 | 1.27 | -1.97 | 0.049 |
|  |  | Unequal x 8&9-year-olds | -2.76 | 1.62 | -1.70 | 0.088 |
|  | Distribution x Decider gender | Unequal x Male | 1.99 | 1.24 | 1.60 | 0.109 |
| Advantageous Inequity |  | Intercept | 2.80 | 0.47 | 5.90 | < 0.001 |
|  | Distribution | Unequal | 0.47 | 0.59 | 0.80 | 0.424 |
|  | Age group | 6&7-year-olds | -0.82 | 0.61 | -1.34 | 0.179 |
|  |  | 8&9-year-olds | -0.46 | 0.63 | -0.74 | 0.462 |
|  | Distribution x Age group | Unequal x 6&7-year-olds | -0.39 | 0.71 | -0.56 | 0.578 |
|  |  | Unequal x 8&9-year-olds | -2.36 | 0.70 | -3.37 | < 0.001 |
